# Factors Influencing the Outcome of Symptomatic Intracranial Artery Stenosis With Hemodynamic Impairment After Short and Long-Term Stent Placement

**DOI:** 10.3389/fneur.2022.682694

**Published:** 2022-05-17

**Authors:** Wentao Gong, Xianjun Zhang, Zhen Meng, Feifei Liu, Guangwen Li, Juan Xiao, Peng Liu, Yujie Sun, Tonghui Liu, Hongxia Wang, Yong Zhang, Naidong Wang

**Affiliations:** ^1^Department of Neurology, The Affiliated Hospital of Qingdao University, Qingdao, China; ^2^Department of Interventional Operating Room, The Affiliated Hospital of Qingdao University, Qingdao, China; ^3^Department of General Medicine, The Affiliated Hospital of Qingdao University, Qingdao, China; ^4^Department of Evidence-Based Medicine, The Second Hospital of Shandong University, Jinan, China

**Keywords:** hemodynamic impairment, intracranial stenosis, stent, risk factor, prognosis

## Abstract

**Objective:**

Stent placement is a feasible approach worldwidely for patients with symptomatic intracranial artery stenosis (sICAS) and hemodynamic impairment (HI) who are at high risk of recurrent stroke after medical treatment. Exploration of factors associated with poor outcomes after stent placement could help develop better individualized therapeutic strategies.

**Methods:**

This study conducted a *post-hoc* analysis of a prospective, multicenter registry study of stent use for sICAS with HI in China. Patient and clinical demographics, and stenotic lesion images were analyzed using univariate and multivariate Cox regression to the time until any endpoints or the end of the follow-up period. The short-term endpoint included any transient ischemic attack (TIA), stroke, or death within 1 month after stent placement. The long-term endpoints included the short-term endpoints and any TIA or stroke in the region of the affected artery that occurred more than 1 month after stent placement.

**Results:**

Two hundred and ninety two patients were included, with 13 short-term and 39 long-term endpoints. Multivariate Cox regression analysis revealed that lesions at the arterial origin or bifurcation (Hazard Ratio (HR) = 7.52; 95% CI, 1.89–29.82; *p* = 0.004) were significantly associated with higher short-term risk. Baseline renal insufficiency reduced the risk (HR = 0.08; 95% CI: 0.01–0.68; *p* = 0.021). Factors significantly associated with higher long-term risk included irregular or ulcerated plaques at the lesion (HR = 2.15; 95% CI: 1.07–4.33; *p* = 0.031). Subgroup analyses indicated that higher risk occurred in the older age group (age>59 years, HR = 3.73, 95% CI: 1.27–10.97, *p* = 0.017), and not in the younger group (age≤59 years, HR = 1.12, 95% CI: 0.42–3.03, *p* = 0.822).

**Conclusion:**

Irregular or ulcerated plaques in older patients and lesions at the arterial opening or bifurcation were more likely to result in adverse endpoints for stent placement during long or short -term follow-up. Investigation of these factors might facilitate the development of individualized therapeutic strategies for this population.

**Clinical Trial Registration:**

http://www.clinicaltrials.gov, identifier: NCT01968122.

## Introduction

Atherosclerotic intracranial stenosis is a major cause of ischemic stroke, especially in the Asian population, where approximately 40% of ischemic strokes are due to intracranial artery stenosis (1). Recently, the predominant treatment for sICAS includes medical and endovascular treatments, of which the primary endovascular treatment is the use of stents. Based on the results published from the SAMMPRIS(Stenting and Aggressive Medical Management for Preventing Recurrent Stroke in Intracranial Stenosis) (2) and VISSIT(Vitesse Intracranial Stent Study for Ischemic Therapy) (3) studies, medical treatment has been the preferred option for sICAS.

However, patients with hypoperfusion-related stroke due to ICAS are at high risk for recurrent stroke even after adequate antithrombotic and vascular risk factor management (4–8). It has been reported that hypoperfusion-related stroke accounts for more than 50% of all sICAS-related strokes that occur in the anterior circulation (9). Endovascular treatment can improve hypoperfusion by reducing the degree of stenosis at the lesion and decreasing the occurrence of additional ischemic events, which might benefit patients more than medicinal treatments. Therefore, this group of patients gained increased attention as being potentially suitable for endovascular treatment.

Our previous study demonstrated that the effectiveness and safety of endovascular therapy using stents in patients with sICAS and HI was acceptable during the 30-day perioperative period and 1-year postoperative follow-up (10, 11). Nevertheless, nearly 10% of the patients experienced stroke or death up to 1 year after stent placement (11). Even higher rates were observed when target artery-related TIAs were included or when the follow-up time was longer, especially in Asian, African-American, and Hispanic populations (1, 11, 12).

Therefore, we explored relevant risk factors affecting short-term and long-term outcomes after the use of stents to treat sICAS with HI. Patient demographic data, vascular risk factors, as well as lesion and procedure-specific characteristics were assessed to establish a baseline. We anticipated that risk factor assessment would allow the identification of a high-risk group and develop individualized treatment plans for those patients. Such treatment could improve the overall favorable prognosis of patients after endovascular treatment.

## Materials and Methods

### Study Design and Subjects

The protocols and detailed outcomes of the prospective, multicenter registry trial in China for the use of stents have been published previously (13). This prospective study evaluated the real-world safety and efficacy of stent use in patients with severe sICAS and HI during the perioperative and long-term follow-up periods in China after the SAMMPRIS trial. HI was defined as the presence of one of the following events. (1) Computed tomography (CT) perfusion imaging revealed a reduction in cerebral blood flow that was 30% compared with the contralateral region. (2) The score for ASITN/SIR on digital subtraction angiography (DSA) was <3 (14). (3) The stroke was caused by hemodynamic abnormalities in the qualifying area (e.g., a watershed cerebral infarction). (4) Single-photon emission CT demonstrated hypoperfusion in the cerebral region in the qualifying area. (5) Transcranial Doppler revealed that the peak blood flow velocity was 200 cm/s during systole. Written informed consent was obtained from all patients or their legal proxy. Eight patients were excluded from the study due to incomplete clinical baseline information. Therefore, 292 patients were included in the risk-factor analysis in this study.

### Composite Endpoint Events

The short-term endpoints included any TIA, stroke, or death within one month after stent placement. The long-term endpoints included the short-term endpoints and any TIA or stroke in the region of the artery with the stent that occurred more than 30 days following stent placement. TIA was defined as any sudden, temporary, and reversible onset of neurological dysfunction that occurred within 24 h after focal cerebral ischemia. Stroke was classified as ischemic stroke, defined as a new neurological deficit persisting for more than 24 h and confirmed by brain CT or magnetic resonance imaging (MRI) or hemorrhagic stroke, including hemorrhage distributed in the brain parenchyma, subarachnoid space, or ventricles.

### Individualized Stent Treatment

Individualized surgical plans were made by the surgeon in each center. The specific patient and lesion characteristics were determined using the registry trial protocol. The focus of consideration for selecting stent types included proximal artery access and lesion morphology.The self-expanding stent (Wingspan Stent System, Stryker, Maple Grove, Minnesota, USA) was the first choice for Mori B or C lesions, a more tortuous access, or a mismatch of the proximal and distal reference lesion diameters. The balloon mounted stent(Apollo stent system, MicroPort Medical, Shanghai, China) was preferred for Mori A lesions and smoother arterial access.

### Medication Regimens and Vascular Risk Factor Management

The medical and risk-factor control protocol development was based on the SAMMPRIS study (2). Preoperative dual antiplatelet therapy (aspirin 100 mg and clopidogrel 75 mg daily) lasted at least 5 days. However, a loading dose of clopidogrel was used when the antiplatelet therapy lasted <5 days. The dual antiplatelet regimen was used at least 3 months after stent placement. As to the vascular risk factor management in long-term follow-up, the systolic blood pressure target was <140 mmHg (130 mmHg for diabetic patients) and the management target for low-density lipoprotein was <70 mg/dl (1.8 mmol/L) or a 50% reduction from the preoperative level. Blood glucose levels, lifestyle factors (body mass index (BMI), smoking cessation, and moderate exercise) were monitored and managed at follow-up examinations.

### Study Factors

The study factors are shown in Supplementary Tables 1, 2 and classified as baseline demographics, clinical features, and stenotic lesion images. Plaque surface morphology (PSM) was evaluated and confirmed by DSA, a subjective evaluation without specified criteria. This evaluation method was based on a previous publication (15).

### Statistical Methods and Analysis

We applied the Kolmogorov-Smirnov test to assess the normality of the continuous variable distribution. Variables that conformed to a normal distribution were expressed as means±SD. Other results were expressed as medians (interquartile ranges, IQRs). Age, degree of stenosis, time of qualifying event (QE) to stent and hemoglobin level were divided by the median and the cut-off value for the NIHSS score, lesion length, level of low-density lipoprotein cholesterol (LDL-c), creatinine clearance (Ccr), and fasting blood glucose (FBG), based on clinical significance.

Cox proportional hazards regression analysis was used to evaluate the correlation between the endpoints and the baseline variables. Before applying the Cox regression analysis, we verified the assumption of proportional hazards using log-minus-log plots for dichotomous variables and Schoenfeld residual analysis for continuous variables. No violations were observed. First, each variable was included in the Cox proportional hazards model for univariate analysis. The inclusion criteria for variables in the multivariate analysis were as follows. (1) A p-value that was 0.200 or less in the univariate analysis of each variable(2) The covariates were screened based on clinical experience and previous results reported in similar published studies (16, 17). The backward elimination method was used to filter the variables and establish the final model.

The sensitivity analysis was carried out as follows. (1) For the short and long-term endpoints, an alternative approach to model construction (Change-In-Estimate) was applied to test the reliability of the results (18). The independent variables were screened for inclusion in the multivariate analysis using the principles described above. Subsequently, any non-significant variables in the results were excluded from the model unless the removal of the variable caused a 10% or greater change in the HR values of other variables in the model. (2) We analyzed non-stroke deaths as competing events in a competing risk model to compare the HR changes with the Cox regression analysis of the significant risk factors for the long-term endpoints.

We analyzed the interaction effects of the plaque surface morphology (PSM) with other variables and performed subgroup analyses to examine whether the potential association between PSM and the long-term endpoints was altered by other variables (19). The interactions among the subgroups were examined using the Cox proportional hazards model. The significance of p for interaction was determined by the likelihood ratio test.

A two-sided *P*-value <0.05 was considered statistically significant. All analyses were performed using SPSS statistical software version 26.0 (SPSS Inc., Chicago, IL, USA) and R statistical software version 4.0.3 (R Foundation).

## Results

### Short and Long-Term Outcomes

Of the 292 patients, a short-term endpoint occurred in 13 patients with a median follow-up time of 31 days (IQR 30–34). There were seven cases of ischemic stroke, five cases of TIA, and one case of hemorrhagic stroke. When the study was completed, the median follow-up time was 24.27 months (IQR 18.77–28.03). The long-term endpoint occurred in 39 cases, including 18 cases of ischemic stroke, 20 cases of TIA, and one case of hemorrhagic stroke. No multiple endpoint events were identified in the same patient during the follow-up period.

### Univariate Analysis

The results of the univariate analysis of the short-term and long-term endpoints are shown in Supplementary Tables 1, 2. The modified Rankin scale (mRS) scores were associated with short-term endpoints. No correlations were observed for any other variables. The incidence of short-term endpoints was 1.1% (1/93) for mRS <1, and 6.0% (12/199) for mRS ≥1 (*p* = 0.09). Concerning long-term endpoints, we obsevered a significant association with plaque surface morphology (PSM) (Supplementary Figures 1, 2), with three (2.3%) events in the smooth surface group and ten (6.3%) events in the irregular or ulcerated surface group.

### Multivariate Analysis

We performed a filter on the variables described above based on the univariate analysis results (variables with *P* ≤ 0.200) and on clinical experience and previous results observed in similar published reports (16, 17) for the multivariate analysis. The significant factors are showed in Table 1.

**Table 1:** Significant factors for short or long-term outcome in Multivariable Cox regression analyses.

	**HR (95% CI)**	***P*** **-value**
**Short-term outcome**		
Ccr mL/(min·1.73m^2^)	(<90 vs. ≥90) 0.08 (0.01–0.68)	0.021
Lesion location	(origin or bifurcation vs. trunk) 7.52 (1.89–29.82)	0.004
**Long-term outcome**		
PSM	(Irregular or ulcerated vs. smooth) 2.15 (1.07–4.33)	0.031

*HR, hazard ratio; CI, confidence interval; Ccr, creatinine clearance; PSM, plaque surface morphology*.

For the short-term endpoints, the following dichotomous variables were included in the multivariate analysis: age, sex, history of hyperlipidemia, history of diabetes mellitus, history of smoking, mRS score, PSM, degree of stenosis, hemoglobin level, Ccr, eccentric lesion, lesion location (Supplementary Figures 3, 4) and length, time of QE to stent, and antiplatelet load. In the final Cox regression model obtained by applying the backward elimination method, the lesion location with a higher risk for lesions located at origins or bifurcations (HR = 7.52, 95% CI:1.89–29.82, *P* = 0.004) and the Ccr level (lower risk of Ccr≤90 mL/(min·1.73 m^2^), HR = 0.08, 95% CI:0.01–0.68, *P* = 0.021) were statistically significant risk factors.

For the long-term endpoints, the following dichotomous variables, except for the degree of residual stenosis, were included in the multivariate analysis: age, sex, history of hyperlipidemia, history of diabetes mellitus, history of hypertension, history of smoking, creatinine clearance, body mass index (BMI), mRS score, anesthesia type, PSM, lesion location, eccentric lesion, degree of stenosis, and residual stenosis. A stepwise Cox regression using the method of backward elimination was conducted to construct the final model. Only PSM was a valid predictor for the long-term endpoint. The presence of irregular or ulcerated plaques increased the long-term endpoint risk compared to smooth plaques (17.6% vs. 8.3%, HR = 2.15, 95% CI:1.07–4.33, *P* = 0.031).

### Sensitivity Analysis

#### Short-Term Risk Factors

**Figure 1:**
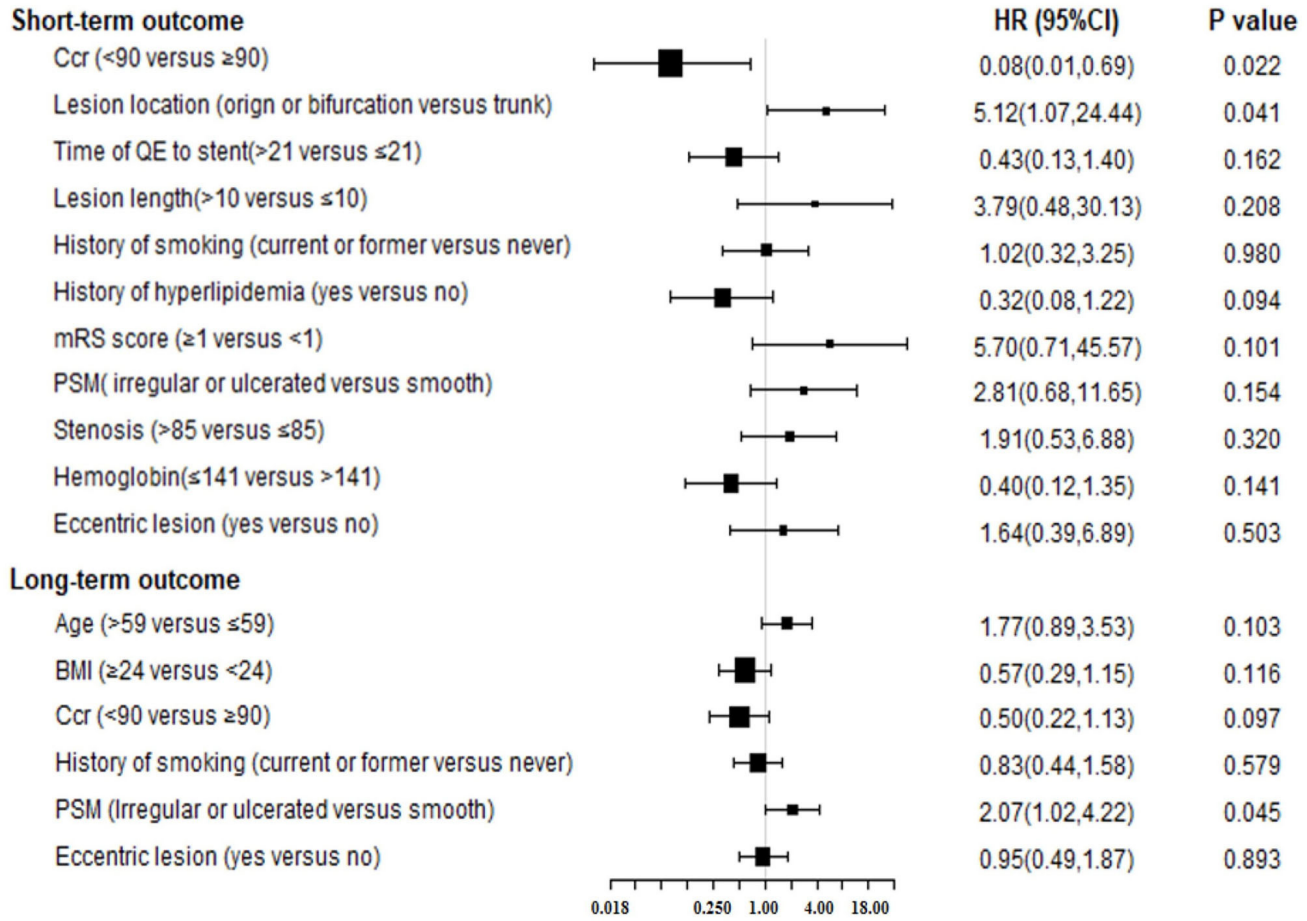
The forest plot for results of Cox multivariable analysis for short or long-term outcome by “change-in-estimate” method. Ccr, creatinine clearance; QE, qualifying event; PSM, plaque surface morphology; BMI, body mass index; HR, hazard ratio; CI, confidence interval.

The variables included in the multivariate analysis also were used to construct the final model using the “change-in-estimate” method (Table 2, Figure 1). The Ccr level was statistically significant (lower risk of Ccr ≤ 90 mL/(min·1.73 m^2^), HR = 0.08, 95% CI:0.01–0.69, *P* = 0.022) as was the lesion location, with a higher risk of lesions located at origins or bifurcations (HR = 5.12, 95% CI:1.07–24.44, *P* = 0.041).

**Table 2:** Results of Cox multivariable analysis for short or long-term outcome by “change-in-estimate” method.

	**HR (95% CI)**	***P*** **-value**
**Short-term outcome**		
Ccr (<90 vs. ≥90)	0.08 (0.01–0.69)	0.022
Lesion location (origin or bifurcation vs. trunk)	5.12 (1.07–24.44)	0.041
Time of QE to stent (>21 vs. ≤ 21)	0.43 (0.13–1.40)	0.162
Lesion length(>10 vs. ≤ 10)	3.79 (0.48–30.13)	0.208
History of smoking (current or former vs. never)	1.02 (0.32–3.25)	0.980
History of hyperlipidemia (yes vs. no)	0.32 (0.08–1.22)	0.094
mRS score (≥1 vs. <1)	5.70 (0.71–45.57)	0.101
PSM (Irregular or ulcerated vs. smooth)	2.81 (0.68–11.65)	0.154
Stenosis (>85 vs. ≤ 85)	1.91 (0.53–6.88)	0.320
Hemoglobin (≤ 141 vs. >141)	0.40 (0.12–1.35)	0.141
Eccentric lesion (yes vs. no)	1.64 (0.39–6.89)	0.503
**Long-term outcome**		
Age (>59 vs. ≤ 59)	1.77 (0.89–3.53)	0.103
BMI (≥24 vs. <24)	0.57 (0.29–1.15)	0.116
Ccr (<90 vs. ≥90)	0.50 (0.22–1.13)	0.097
History of smoking (current or former vs. never)	0.83 (0.44–1.58)	0.579
PSM (Irregular or ulcerated vs. smooth)	2.07 (1.02–4.22)	0.045
Eccentric lesion (yes vs. no)	0.95 (0.49–1.87)	0.893

#### Long-Term Risk Factors

**Figure 2:**
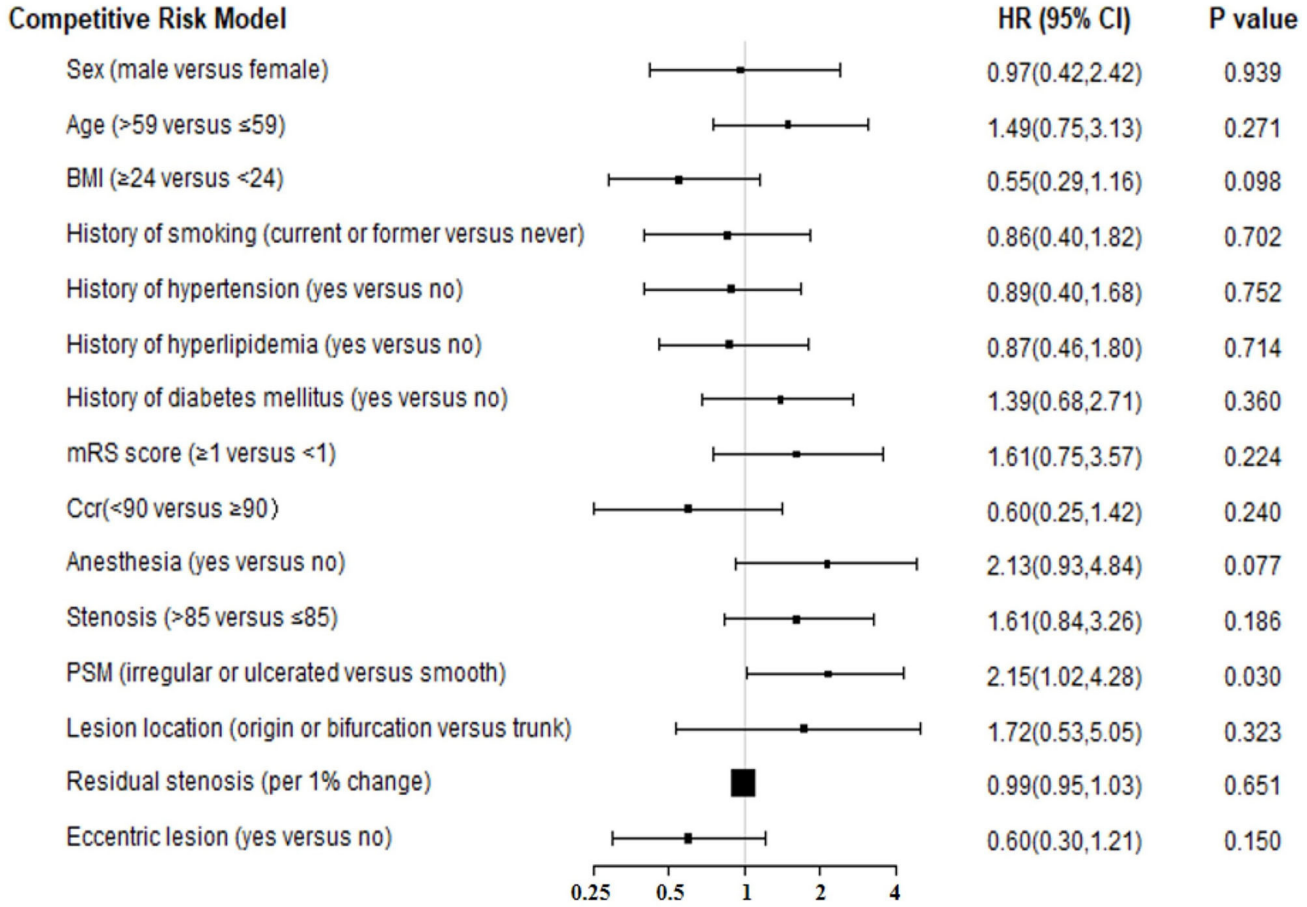
The forest plot for results of competitive risk model for long-term outcome. BMI, body mass index; Ccr, creatinine clearance; PSM, plaque surface morphology; HR, hazard ratio; CI, confidence interval.

The “change-in-estimate” method was used to assess the long-term events existed in (Table 2, Figure 1). The results from the final model revealed that PSM, specifically irregular or ulcerated plaques, was the only significant predictor (HR = 2.07, 95% CI:1.02–4.22, *P* = 0.045). Concerning the competitive risk model analysis, we included the variables described above and repeated the multivariate analysis using a competitive risk model (Table 3, Figure 2). Three competing events were included, two for malignancy-related deaths and one for unexplained deaths. The results demonstrated that PSM was significant (HR = 2.15, 95% CI:1.08–4.27, *P* = 0.030).

**Table 3:** Results of competitive risk model for long-term outcome.

	**HR (95% CI)**	***P*** **-value**
Sex (Male vs. female)	0.97 (0.40–2.35)	0.939
Age (>59 vs. ≤ 59)	1.49 (0.73–3.04)	0.271
BMI (≥24 vs. <24)	0.55 (0.27–1.12)	0.098
History of smoking (Current or former vs. never)	0.86 (0.40–1.85)	0.702
History of hypertension (yes vs. no)	0.89 (0.43–1.85)	0.752
History of hyperlipidemia (yes vs. no)	0.87 (0.42–1.81)	0.714
History of diabetes mellitus (yes vs. no)	1.39 (0.68–2.85)	0.360
mRS score (≥1 vs. <1)	1.61 (0.75–3.45)	0.224
Ccr (<90 vs. ≥90)	0.60 (0.25–1.42)	0.240
Anesthesia (yes vs. no)	2.13 (0.92–4.94)	0.077
Stenosis (>85 vs. ≤ 85)	1.61 (0.79–3.28)	0.186
PSM (Irregular or ulcerated vs. smooth)	2.15 (1.08–4.27)	0.030
Lesion location (origin or bifurcation vs. trunk)	1.72 (0.59–5.06)	0.323
Residual stenosis (per 1% change)	0.99 (0.95–1.03)	0.651
Eccentric lesion (yes vs. no)	0.60 (0.30–1.21)	0.150

### Interaction Effects and Subgroup Analysis

**Figure 3:**
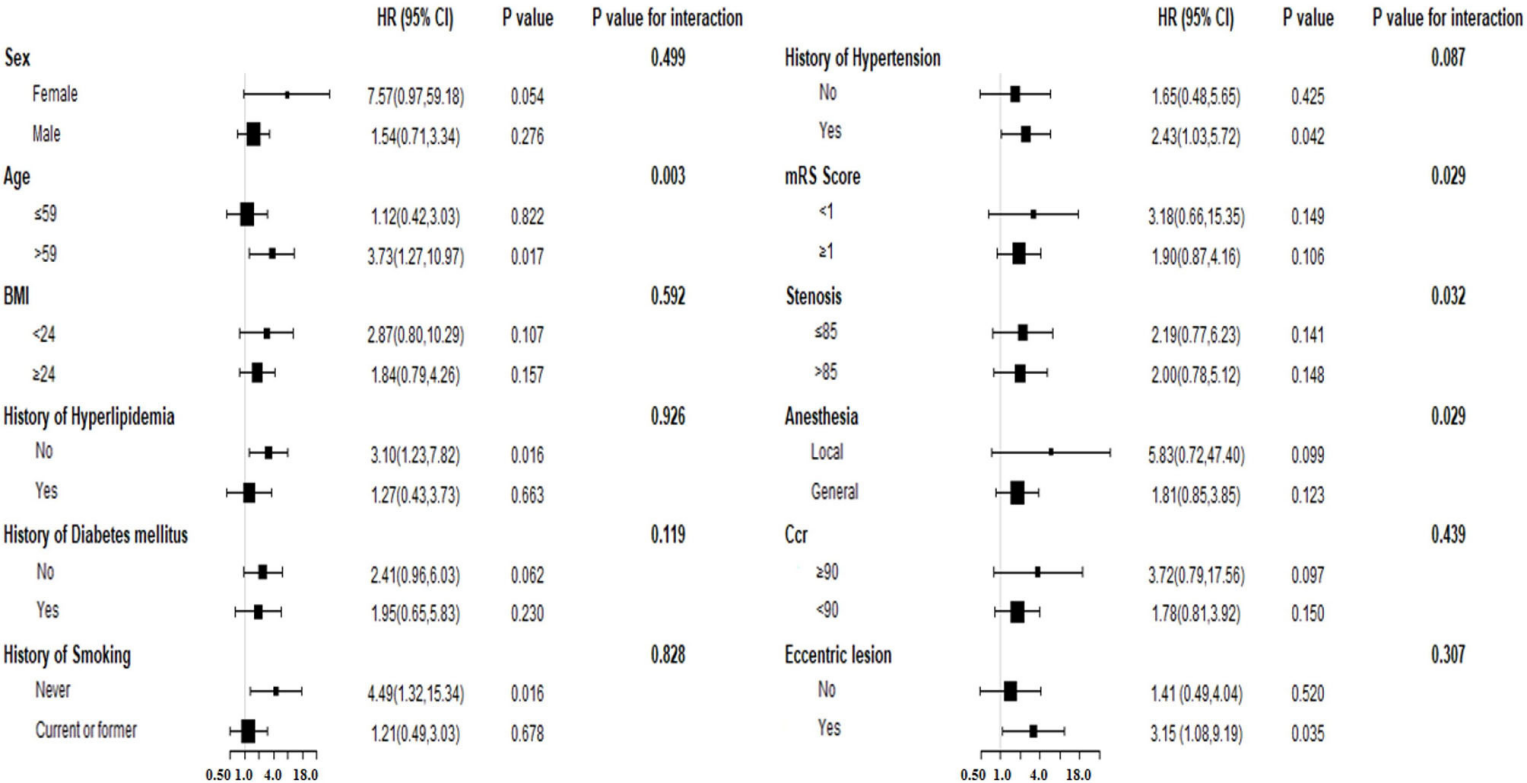
The forest plot for subgroup analysis for association between PSM and long-term events. BMI, body mass index; Ccr, creatinine clearance; PSM, plaque surface morphology; HR, hazard ratio; CI, confidence interval.

Concerning the significant risk factor for long-term follow-up, we explored whether PSM exhibited an interaction effect with other variables. We stratified the participants based on the following variables: age, sex, history of hyperlipidemia, history of diabetes mellitus, history of hypertension, history of smoking, BMI, mRS score, anesthesia type, eccentric lesion, Ccr, and degree of stenosis, which were the variables included in the multivariate analysis and the significant short-term risk factor (Ccr). The results of the analysis (Table 4, Figure 3) indicated an interaction effect between PSM and age (p=0.003 for the interaction). A higher long-term endpoint risk existed in the presence of irregular or ulcerated plaques in the older population (older than 59 years, HR = 3.73, 95%CI:1.27–10.97, *p* = 0.017). The significance did not change after further adjustment for the above variables and the degree of residual stenosis. No significant differences were observed for the risk in middle-aged and young adults (age ≤ 59, HR = 1.12, 95%CI:0.42–3.03, *p* = 0.822).

**Table 4:** Subgroup analysis for association between PSM and long-term events.

	**PSM** **(Irregular or ulcerated vs. smooth)**	***P*** **-value**	***P*** **-value for interaction**
	**HR (95% CI)**		
Sex			0.499
Female	7.57 (0.97–59.18)	0.054	
Male	1.54 (0.71–3.34)	0.276	
Age (median, y)			0.003
≤ 59	1.12 (0.42–3.03)	0.822	
>59	3.73 (1.27–10.97)	0.017	
BMI			0.592
<24	2.87 (0.80–10.29)	0.107	
≥24	1.84 (0.79–4.26)	0.157	
History of hyperlipidemia			0.926
No	3.10 (1.23–7.82)	0.016	
Yes	1.27 (0.43–3.73)	0.663	
History of diabetes mellitus			0.119
No	2.41 (0.96–6.03)	0.062	
Yes	1.95 (0.65–5.83)	0.230	
History of smoking			0.828
Never	4.49 (1.32–15.34)	0.016	
Current or former	1.21 (0.49–3.03)	0.678	
History of hypertension			0.087
No	1.65 (0.48–5.65)	0.425	
Yes	2.43 (1.03–5.72)	0.042	
mRS Score			0.029
<1	3.18 (0.66–15.35)	0.149	
≥1	1.90 (0.87–4.16)	0.106	
Stenosis (%, median)			0.032
≤ 85	2.19 (0.77–6.23)	0.141	
>85	2.00 (0.78–5.12)	0.148	
Anesthesia			0.029
Local	5.83 (0.72–47.40)	0.099	
General	1.81 (0.85–3.85)	0.123	
Ccr mL/(min·1.73m^2^)			0.439
≥90	3.72 (0.79–17.56)	0.097	
<90	1.78 (0.81–3.92)	0.150	
Eccentric lesion			0.307
No	1.41 (0.49– 4.04)	0.520	
Yes	3.15 (1.08–9.19)	0.035	

## Discussion

Currently, few published studies have assessed the short-term and long-term risk factors associated with stent use in patients with sICAS and HI. This study identified risk factors associated with combined sICAS and HI to make endovascular therapy safer and more effective.

Previous studies on short-term risk factors associated with intracranial artery stent placement were reported in the SAMMPRIS study. Fiorella et al. determined that factors associated with perioperative ischemic events included nonsmoking, basilar artery stenosis, diabetes mellitus, and increased age (16). Conversely, greater stenosis, low mRS scores, and clopidogrel loading increased the risk for hemorrhagic stroke (16). When stroke and death were defined as a combined endpoint for analysis, only nonsmoking and longer lesions were associated with increased risk, but did not exhibit good predictive values (17). Our findings revealed that lesions located at arterial origins or bifurcations exhibited a higher short-term postoperative risk.

The observed discrepancies were due to several factors. First, the characteristics of the included patients demonstrated differences. We included patients with HI in the region of the qualified artery and excluded individuals with a penetrator stroke (13). However, similar patients accounted for only 50–60% of the individuals with anterior circulation cases included in the SAMMPRIS study (9). Second, differences were existed in the surgical protocols. We individually selected balloon dilated or self-expanding stents based on different vascular access and lesion characteristics. The advantages of the balloon dilated stent (the Apollo stent system) include more accurate stent positioning at the lesion and higher radial support to the vessel compared to the self-expanding stent in relatively straight and short lesions, which results in a lower postoperative luminal residual stenosis rate. However, for Mori C lesions or lesions with a significant mismatch between the proximal and distal diameters, there is a higher risk of arterial dissection and rupture and poor stent apposition after balloon dilated stent release (20). Furthermore, due to its poor flexibility, the balloon-dilated stent often is unable to reach the lesion if the intracranial vessels are tortuous. The self -expanding stent can overcome the shortcomings of the balloon dilated stent due to its greater flexibility, which increases the ability to pass through tortuous vessels. Therefore, the balloon dilated stent is preferable for patients with smooth access and Mori A type lesions while the self-expanding stent (Wingspan stent system) is recommended for complex intracranial lesions (13). We also adopted submaximal angioplasty to decrease the incidence of perioperative complications, which were 4.3% vs. 14.7% in the final results obtained within 30 days in SAMMPRIS study (2, 10). Other differences included small overall sample sizes, relatively few positive events, and hemorrhagic stroke events, which resulted in low statistical power for some candidate risk factors. Finally, the defined endpoint event type in this study differed from the SAMMPRIS study (2).

In our research, short-term adverse events were more prone to occur when the lesion was located at the proximal origin of the artery or a branch artery emanating from it (12.00% vs. 3.75%, adjusted HR = 7.52 *P* = 0.004). Ischemic events occurred in the region of the branch arteries in some patients with a concomitant atherosclerotic stenosis in the trunk-branch arterial junction (21). These events are due to endothelial damage, dissection, plaque displacement, or the stent scaffolding shielding that resulted in increased stenosis or occlusion (20), especially with poor collateral circulation. Despite the availability of numerous branch protection techniques, bifurcation lesions continue to present challenges and a higher risk for interventional procedures (22).

When endovascular treatment is performed for an origin lesion, inadequate balloon dilation, high residual stenosis, or poor stent apposition will occur because most balloons and stents cross the inter-arterial angles (ICA-MCA, VA-BA) (21). It is challenging to select the appropriate stent size when referring to the proximal lumen diameter (21).

Renal insufficiency is often associated with a poor prognosis after stroke (23). Surprisingly, renal insufficiency was a protective factor for short-term outcomes (adjusted HR for Ccr ≤90 mL/(min·1.73 m^2^)=0.08, *P* = 0.021). Over 90% (12/13) of the short-term adverse outcomes were ischemic events. Activation of the local coagulation system due to endothelial and plaque injury after stent placement produces acute or subacute thrombosis at the lesion (24, 25), which could lead to occlusion of the penetrating or branch artery adjacent to the lesion and embolization of the distal cortical artery. In patients with renal insufficiency, the impairment in hemostasis causes a certain degree of inhibition of the process of activation of the coagulation system and the thrombosis thereby reducing the risk of short-term ischemic events. The reasons include: (1) the defect in platelet secretion, adhesion, secretion and platelet-vessel wall interaction (26); (2) reduced activity of some coagulation factors and sustained activation of the fibrinolytic system (27). However, other studies have shown that patients with chronic kidney disease are less responsive to antiplatelet agents and more likely to develop aspirin or clopidogrel resistance (28–30), further increasing the risk of post-PCI adverse events (31). The actual platelet function and risk of post-stent ischemia in patients with or without combined renal insufficiency are still unclear and need additional investigation.

In our study, the low level of baseline creatinine clearance in some patients could not be excluded as acute kidney injury. Therefore, this index did not reflect the patient's long-term preoperative renal functional status. Also, contrast-associated acute kidney injury should not be ignored, as it is more likely to occur in patients with renal insufficiency (32). Therefore, the safety of stent use in patients with renal insufficiency needs additional evaluation.

We discovered that PSM was a predictor of long-term adverse events, and it was correlated with adverse events in the short-term outcome analysis. Irregularities in the luminal surface of carotid plaques or the presence of ulcerative lesions are strongly associated with ischemic stroke episodes (33). Even when carotid stent placement was performed, some studies have shown that DSA-proven carotid-ulcerated plaques still increase the risk of stroke at 30 days after carotid stent placement and is an independent predictor of a new ipsilateral stroke (34, 35). Nevertheless, information is limited on whether such plaque characteristics affect the long-term prognosis after carotid stent placement and stent placement in intracranial arteries.

Surface irregularities or ulcerations often are features of unstable plaques (15). These features are thought to reflect endothelial dysfunction and the systemic and local plaque chronic inflammatory state (36, 37). Inflammation can further induce in-stent neoatherosclerosis and late thrombosis due to incomplete post-procedure endothelialization and persistent chronic inflammatory cell infiltration (38, 39). An observational study after coronary stent placement found that neoatherosclerosis was associated with an increased incidence of long-term major adverse cardiac events (HR = 2.909, *P* = 0.012) (40). We speculated that the reasons mentioned above might be important for the poor long-term prognosis after stent placement due to specific plaque morphologies.

Subgroup analysis revealed that the effect of irregular or ulcerated plaques on long-term adverse events varied across age groups. Older adults with these plaque features exhibited high long-term risk following stent placement. A previous study suggested that older adults were more prone to ischemic events within 30 days of the perioperative period after intracranial stent placement (16). However, the relevance at longer follow-up times has not been confirmed. Older individuals exhibit higher systemic inflammatory activity, which is, in part, related to the numerous chronic diseases associated with age (41). On the other hand, somatic mutations associated with biological aging promote the development of atherosclerosis (42). These factors complicate normal repair of the vessel wall after stent placement and accelerate neoatherosclerosis formation, which results in an unfavorable long-term prognosis.

Our study did not reveal any significant association between residual stenosis and long-term outcomes in either the COX regression (HR = 1.00, 95% CI: 0.96–1.04, *P* = 0.886) or the competing risk model (HR = 0.99, 95% CI: 0.95–1.03, *P* = 0.651). A *post-hoc* analysis from the SAMMPRIS study revealed that symptomatic in-stent restenosis (ISR) was the leading cause of cerebral infarction during the postoperative follow-up period (43). Several other studies have reported that higher postoperative residual stenosis might be a risk factor for ISR during the follow-up period, particularly when the residual stenosis is >30% (44, 45), because higher residual stenosis might indicate an inadequately treated lesion. In our registry study, there were eightcases with residual stenosis ≥30%, accounting for only 2.8% of the total sample number. Therefore, in this sample, residual stenosis only had a small effect on ISR, which might be one reason why residual stenosis was not associated with any long-term outcomes. Another analysis from our registry study showed no significant difference in the degree of postoperative residual stenosis between patients in the ISR group compared to the non-ISR group, and that residual stenosis was not a risk factor for ISR (46). However, the results of a meta-analysis suggested that lower residual stenosis increased the risk of in-stent restenosis (47). The authors concluded that a lower residual stenosis indicated the occurrence of aggressive dilation and more severe endothelial injury (47). Therefore, there migh be a U-shaped rather than linear relationship between residual stenosis and ISR or even the long-term outcomes. The linear regression analysis applied in our study may not reveal this U-shaped relationship and the above findings need to be confirmed by additional studies.

Our analysis of the relevant risk factors could improve individualized protocols for intracranial stent use in patients with sICAS with HI. When the lesion is located at the origin or bifurcation of the artery and significant branch arteries are present, we need to carefully estimate the difficulty and risk of the procedure before and during stent placement. With the occurrence of irregular or ulcerated plaques in older patients, the preferred treatment protocol should focus on systemic factors, which might not be limited to the traditional risk factors associated with cerebrovascular disease (41). Also, risk evaluation for recurrence should continue throughout the entire follow-up period. Finally, changes in the treatment modality to address the high-risk lesion characteristics mentioned above, with the option of balloon angioplasty alone when appropriate, could improve the safety and effectiveness of treatment even further (48–50).

We noted several limitations in this study. First, multiple adverse events were defined as a composite clinical outcome, which ignored the influence of each event on the patient's prognosis. Therefore, our risk predictors might not accurately reflect the actual patient survival after the event. Second, the application of five criteria to evaluate HI resulted in heterogeneity in the hemodynamic status among the enrolled patients, which weakened the population representation. Third, the classification of plaque surface morphology by DSA was subjective. Future studies could combine CTA, high-resolution MRI, and other assessment modalities to allow a more accurate determination of plaque surface morphology. Finally, although this study presented the largest sample size among similar studies, it was a *post hoc* analysis. There, which could have underestimated the relationships between some variables and risk. There were challenges in establishing an accurate and comprehensive regression model. Thus, we used other modeling methods for sensitivity analysis to confirm the reliability of the results. Additional clinical studies with larger sample sizes are needed to validate these results.

## Conclusion

We reported that in patients with sICAS combined with HI, the higher risk of short-term follow-up was primarily associated with preoperative lesions located at arterial origins or bifurcations, and irregular or ulcerated plaques also might be related to higher short-term risk. Higher risk at long-term follow-up also was associated with irregular or ulcerated plaques. When considering the presence of short-term or long-term risk factors, a poor prognosis after stent placement should be considered, and other rational individualized treatment strategies need to be explored.

## Data Availability Statement

The raw data supporting the conclusions of this article will be made available by the authors, without undue reservation.

## Ethics Statement

The studies involving human participants were reviewed and approved by the Medical Ethics Committee of the Affiliated Hospital of Qingdao University. The patients/participants provided their written informed consent to participate in this study. Written informed consent was obtained from the individual(s) for the publication of any potentially identifiable images or data included in this article.

## Author Contributions

WG, NW, and YZ were involved in the conception. WG drafted the manuscript. XZ, FL, JX, PL, and HW contributed to the manuscript drafting and revision. TL and YS were involved in the interpretation of data. ZM collected the data images. NW, GL, and YZ revised the manuscript. All authors contributed to the article and approved the submitted version.

## Conflict of Interest

The authors declare that the research was conducted in the absence of any commercial or financial relationships that could be construed as a potential conflict of interest.

## Publisher's Note

All claims expressed in this article are solely those of the authors and do not necessarily represent those of their affiliated organizations, or those of the publisher, the editors and the reviewers. Any product that may be evaluated in this article, or claim that may be made by its manufacturer, is not guaranteed or endorsed by the publisher.

## Abbreviations

ASITN/SIR: American Society of Intervention and Therapeutic Neuroradiology/Society of Interventional Radiology
BA: basilar artery
BMI: body mass index
Ccr: creatinine clearance
CI: confidence interval
CT: computed tomography
DSA: digital subtraction angiography
FBG: fasting blood glucose
HDL-c: high-density lipoprotein cholesterol
HI: hemodynamic impairment
HR: hazard ratio
ICA: internal carotid artery
IQR: interquartile range
LDL-c: low-density lipoprotein cholesterol
MCA: middle cerebral artery
mRS: modified Rankin scale
PSM: plaque surface morphology
QE: qualifying event
sICAS: symptomatic intracranial artery stenosis
TIA: transient ischemic attack
VA: vertebral artery.
