# Supplementary material for: Factors Influencing the Outcome of Symptomatic Intracranial Artery Stenosis With Hemodynamic Impairment After Short and Long-Term Stent Placement
Source: Front Neurol. 2022 May 17;13:682694. doi: 10.3389/fneur.2022.682694 (PMC9152452; doi:10.3389/fneur.2022.682694)
Supplement: Supplementary file 1 [file Data_Sheet_1.docx]

Supplementary Material

Table 1 Difference in categorical variables for short and long-term outcomes on univariate Cox regression analyses

|  |  | Short-term  Outcome | HR | P value | Long-term  Outcome | HR | P value |
| --- | --- | --- | --- | --- | --- | --- | --- |
| Factor | No. | No.(%) | (95% CI) |  | No.(%) | (95% CI) |  |
| Sex |  |  |  |  |  |  |  |
| Female | 73 | 5(6.8%) | 0.52(0.17-1.59) | 0.253 | 11(15.1%) | 0.80(0.40-1.61) | 0.529 |
| Male | 219 | 8(3.7%) |  |  | 28(12.8%) |  |  |
| Age (median, y) |  |  |  |  |  |  |  |
| ≤59 | 148 | 6(4.1%) | 1.21(0.41-3.61) | 0.730 | 16(10.8%) | 1.54(0.82-2.92) | 0.183 |
| ＞59 | 144 | 7(4.9%) |  |  | 23(16.0%) |  |  |
| BMI |  |  |  |  |  |  |  |
| ＜24 | 81 | 5(6.2%) | 0.61(0.20-1.86) | 0.384 | 14(17.3%) | 0.65(0.34-1.26) | 0.200 |
| ≥24 | 211 | 8(3.8%) |  |  | 25(11.8%) |  |  |
| History of Hypertension |  |  |  |  |  |  |  |
| No | 81 | 4(4.9%) | 0.86(0.27-2.80) | 0.805 | 11(13.6%) | 0.93(0.46-1.87) | 0.846 |
| Yes | 211 | 9(4.3%) |  |  | 28(13.3%) |  |  |
| History of Hyperlipidemia |  |  |  |  |  |  |  |
| No | 172 | 10(5.8%) | 0.42(0.12-1.53) | 0.189 | 24(14.0%) | 0.83(0.43-1.59) | 0.576 |
| Yes | 120 | 3(2.5%) |  |  | 15(12.5%) |  |  |
| History of Diabetes mellitus |  |  |  |  |  |  |  |
| No | 206 | 9(4.4%) | 1.06(0.33-3.45) | 0.921 | 25(12.1%) | 1.33(0.70-2.57) | 0.384 |
| Yes | 86 | 4(4.7%) |  |  | 14(16.3%) |  |  |
| History of Smoking |  |  |  |  |  |  |  |
| Never | 133 | 7(5.3%) | 0.71(0.24-2.11) | 0.535 | 20(15.0%) | 0.74(0.40-1.39) | 0.349 |
| Current or former | 159 | 6(3.8%) |  |  | 19(11.9%) |  |  |
| History of Drinking |  |  |  |  |  |  |  |
| No | 178 | 9(5.1%) | 0.69(0.21-2.24) | 0.538 | 27(15.2%) | 0.65(0.33-1.28) | 0.209 |
| Yes | 114 | 4(3.5%) |  |  | 12(10.5%) |  |  |
| History of Stroke or TIA |  |  |  |  |  |  |  |
| No | 153 | 7(4.6%) | 0.94(0.32-2.80) | 0.914 | 20(13.1%) | 1.11(0.59-2.08) | 0.753 |
| Yes | 139 | 6(4.3%) |  |  | 19(13.7%) |  |  |
| QE |  |  |  |  |  |  |  |
| Stroke | 159 | 6(3.8%) | 1.40(0.47-4.16) | 0.546 | 24(15.1%) | 0.76(0.40-1.44) | 0.398 |
| TIA | 133 | 7(5.3%) |  |  | 15(11.3%) |  |  |
| Symptomatic artery |  |  |  | 0.672 |  |  | 0.667 |
| ICA | 40 | 2(5.0%) | Reference | Reference | 7(17.5%) | Reference | Reference |
| MCA | 89 | 3(3.4%) | 0.67(0.11-3.99) | 0.657 | 9(10.1%) | 0.56(0.21-1.51) | 0.253 |
| BA | 93 | 6(6.5%) | 1.31(0.26-6.48) | 0.743 | 14(15.1%) | 0.84(0.34-2.08) | 0.703 |
| VA | 70 | 2(2.9%) | 0.56(0.08-3.99) | 0.564 | 9(12.9%) | 0.69(0.26-1.85) | 0.458 |
| Mori type |  |  |  |  |  |  |  |
| Mori A or B | 237 | 11(4.6%) | 0.77(0.17-3.46) | 0.731 | 33(13.9%) | 0.77(0.32-1.83) | 0.553 |
| Mori C | 55 | 2(3.6%) |  |  | 6(10.9%) |  |  |
| mRS score |  |  |  |  |  |  |  |
| ＜1 | 93 | 1(1.1%) | 5.71(0.74-43.90) | 0.094 | 9(9.7%) | 1.73(0.82-3.64) | 0.151 |
| ≥1 | 199 | 12(6.0%) |  |  | 30(15.1%) |  |  |
| NIHSS score |  |  |  |  |  |  |  |
| ≤1 | 193 | 8(4.1%) | 1.21(0.40-3.71) | 0.735 | 28(14.5%) | 0.74(0.37-1.50) | 0.406 |
| ＞1 | 99 | 5(5.1%) |  |  | 11(11.1%) |  |  |
| Stent type |  |  |  |  |  |  |  |
| balloon mounted | 144 | 5(3.5%) | 1.57(0.51-4.79) | 0.430 | 16(11.1%) | 1.43(0.76-2.71) | 0.273 |
| self-expanding | 148 | 8(5.4%) |  |  | 23(15.5%) |  |  |
| Anesthesia |  |  |  |  |  |  |  |
| Local | 96 | 4(4.2%) | 1.11(0.34-3.61) | 0.860 | 8(8.3%) | 1.80(0.83-3.93) | 0.140 |
| General | 196 | 9(4.6%) |  |  | 31(15.8%) |  |  |
| Antiplatelet load |  |  |  |  |  |  |  |
| No | 263 | 12(4.6%) | 0.74(0.10-5.72) | 0.777 | 36(13.7%) | 0.74(0.23-2.39) | 0.611 |
| Yes | 29 | 1(3.4%) |  |  | 3(10.3%) |  |  |
| Eccentric lesion |  |  |  |  |  |  |  |
| No | 113 | 3(2.7%) | 2.14(0.59-7.79) | 0.247 | 14(12.4%) | 1.11(0.58-2.14) | 0.751 |
| Yes | 179 | 10(5.6%) |  |  | 25(14.0%) |  |  |
| PSM |  |  |  |  |  |  |  |
| Smooth | 133 | 3(2.3%) | 2.87(0.79-10.42) | 0.110 | 11(8.3%) | 2.15(1.07-4.33) | 0.031 |
| Irregular or ulcerated | 159 | 10(6.3%) |  |  | 28(17.6%) |  |  |
| Lesion location |  |  |  |  |  |  |  |
| Trunk | 267 | 10(3.6%) | 3.31(0.91-12.02) | 0.069 | 35(9.54%) | 1.31(0.46-3.68) | 0.612 |
| Origin or Bifurcation | 25 | 3(12.0%) |  |  | 4(16.00%) |  |  |
| Lesion length, mm |  |  |  |  |  |  |  |
| ≤10 | 225 | 12(5.3%) | 0.27(0.04-2.10) | 0.213 | 33(14.7%) | 0.59(0.25-1.41) | 0.237 |
| ＞10 | 67 | 1(1.5%) |  |  | 6(9.0%) |  |  |
| Stenosis (%, median) |  |  |  |  |  |  |  |
| ≤85 | 155 | 4(2.6%) | 2.59(0.80-8.40) | 0.114 | 17(11.0%) | 1.57(0.83-2.95) | 0.165 |
| ＞85 | 137 | 9(6.6%) |  |  | 22(16.1%) |  |  |
| Time of QE to stent (d, median) |  |  |  |  |  |  |  |
| ≤21 | 149 | 8(5.3%) | 0.64(0.21-1.97) | 0.440 | 21(14.1%) | 0.81(0.43-1.53) | 0.521 |
| ＞21 | 143 | 5(3.5%) |  |  | 18(12.6%) |  |  |
| Hemoglobin(g/L, median) |  |  |  |  |  |  |  |
| ≤141 | 148 | 9(6.1%) | 0.45(0.14-1.46） | 0.183 | 21(14.2%) | 0.85(0.45-1.59) | 0.609 |
| ＞141 | 144 | 4(2.8%) |  |  | 18(12.5%) |  |  |
| LDL-c (mmol/L) |  |  |  |  |  |  |  |
| ＜2.6 | 196 | 7(3.6%) | 1.79(0.60-5.33) | 0.295 | 23(11.7%) | 1.51(0.80-2.87) | 0.204 |
| ≥2.6 | 96 | 6(6.3%) |  |  | 16(16.7%) |  |  |
| Ccr mL/(min·1.73m^2^) |  |  |  |  |  |  |  |
| ≥90 | 90 | 1(1.1%) | 0.18(0.02-1.41) | 0.102 | 10(11.1%) | 0.76(0.37-1.57 ) | 0.463 |
| <90 | 202 | 12(5.9%) |  |  | 29(14.4%) |  |  |
| FBG, mmol/L |  |  |  |  |  |  |  |
| ＜7 | 229 | 9(3.9%) | 1.62(0.50-5.27) | 0.420 | 29(12.7%) | 1.23(0.60-2.53) | 0.573 |
| ≥7 | 63 | 4(6.3%) |  |  | 10(15.9%) |  |  |

Abbreviation:

BMI: body mass index QE: qualifying event TIA: transient ischemic attack ICA: internal carotid artery

MCA: middle cerebral artery BA: basilar artery VA: vertebral artery PSM: plaque surface morphology

LDL-c: low-density lipoprotein cholesterol Ccr: creatinine clearance FBG: fasting blood glucose

Table 2 Difference in continuous variables for short and long-term outcomes in univariate Cox regression analyses

| Factor | median (IQR)  or mean ± SD | Short-term Outcome  HR (95% CI) | P value | Long-term Outcome  HR(95% CI) | P value |
| --- | --- | --- | --- | --- | --- |
| Age, y | 59(52-65) | 0.99(0.94-1.05) | 0.732 | 1.02(0.98-1.05) | 0.305 |
| BMI, Kg/m^2^ | 25.56±3.04 | 0.95(0.80-1.14) | 0.602 | 0.98(0.87-1.07) | 0.518 |
| Hemoglobin, g/L | 140.82±14.03 | 0.98(0.94-1.01) | 0.205 | 0.99(0.98-1.02) | 0.818 |
| Neutrophil, % | 63.23±10.66 | 0.99(0.94-1.04) | 0.632 | 1.01(0.98-1.04) | 0.529 |
| Platelet, 10^9^/L | 203(172-243) | 1.00(0.99-1.01) | 0.501 | 1.00(1.00-1.01) | 0.829 |
| Ccr, mL/(min·1.73m^2^) | 105.09±30.75 | 1.01(0.99-1.03) | 0.365 | 1.00(1.00-1.01) | 0.375 |
| FBG, mmol/L | 5.25(4.69-6.72) | 1.05(0.92-1.21) | 0.458 | 1.02(0.93-1.12) | 0.641 |
| Serum uric acid, umol/L | 321.99±86.97 | 1.00(0.99-1.01) | 0.981 | 1.00(1.00-1.00) | 0.535 |
| Triglyceride, mmol/L | 1.39(1.05-2.14) | 0.80(0.44-1.45) | 0.465 | 0.85(0.62-1.16) | 0.301 |
| LDL-c, mmol/L | 2.20(1.63-2.81) | 0.94(0.54-1.64) | 0.834 | 1.11(0.83-1.47) | 0.467 |
| HDL-c, mmol/L | 0.99(0.86-1.19) | 0.49(0.07-3.67) | 0.489 | 0.61(0.22-1.68) | 0.338 |
| Lesion length, mm | 7.16(5.20-9.23) | 1.00(0.83-1.22) | 0.981 | 1.05(0.94-1.17) | 0.374 |
| Normal reference Diameter, mm | 2.90(2.50-3.50) | 0.95(0.49-1.84) | 0.868 | 0.90(0.60-1.34) | 0.588 |
| Time of QE to stenting, d | 21.0(10.00-33.75) | 0.98(0.95-1.02) | 0.320 | 0.99(0.97-1.01) | 0.267 |
| Stenosis, % | 85.0(80.0-90.0) | 1.06(0.98-1.14) | 0.182 | 1.02(0.98-1.07) | 0.282 |
| Residual stenosis, % | 10.0(0-10.0) | 0.99(0.92-1.06) | 0.683 | 1.00(0.96-1.04) | 0.886 |

Abbreviation:

BMI: body mass index Ccr: creatinine clearance FBG: fasting blood glucose

LDL-c: low-density lipoprotein cholesterol HDL-c: high-density lipoprotein cholesterol QE: qualifying eventFigure legends

Supplement Figure legends

Supplement Figure 1.The black arrow points to smooth plaque in the intracranial artery stenosis. A, B, C, and D are the ICA, MCA, VA, and BA respectively. Abbreviation ICA: internal carotid artery MCA: middle cerebral artery BA: basilar artery VA: vertebral artery

Supplement Figure 2 The black arrow points to irregular or ulcerated plaque in the intracranial artery stenosis. A, B, C, and D are the ICA, MCA, VA, and BA respectively. Abbreviation ICA: internal carotid artery MCA: middle cerebral artery BA: basilar artery VA: vertebral artery

Supplement Figure 3 The stenotic lesion indicated by the black arrow are located in the trunk of artery. A, B, C, and D are the ICA, MCA, VA, and BA respectively. Abbreviation ICA: internal carotid artery MCA: middle cerebral artery BA: basilar artery VA: vertebral artery

Supplement Figure 4 The stenotic lesion indicated by the black arrow are located in the opening or bifurcation of artery. A, B, C, and D are the ICA, MCA, VA, and BA respectively. Abbreviation ICA: internal carotid artery MCA: middle cerebral artery BA: basilar artery VA: vertebral artery
