# Supplementary material for: Factors Influencing the Outcome of Symptomatic Intracranial Artery Stenosis With Hemodynamic Impairment After Short and Long-Term Stent Placement
Source: Front Neurol. 2022 May 17;13:682694. doi: 10.3389/fneur.2022.682694 (PMC9152452; doi:10.3389/fneur.2022.682694)
Supplement: Supplementary file 2 [file Presentation_1.PPTX]

## Slide 1
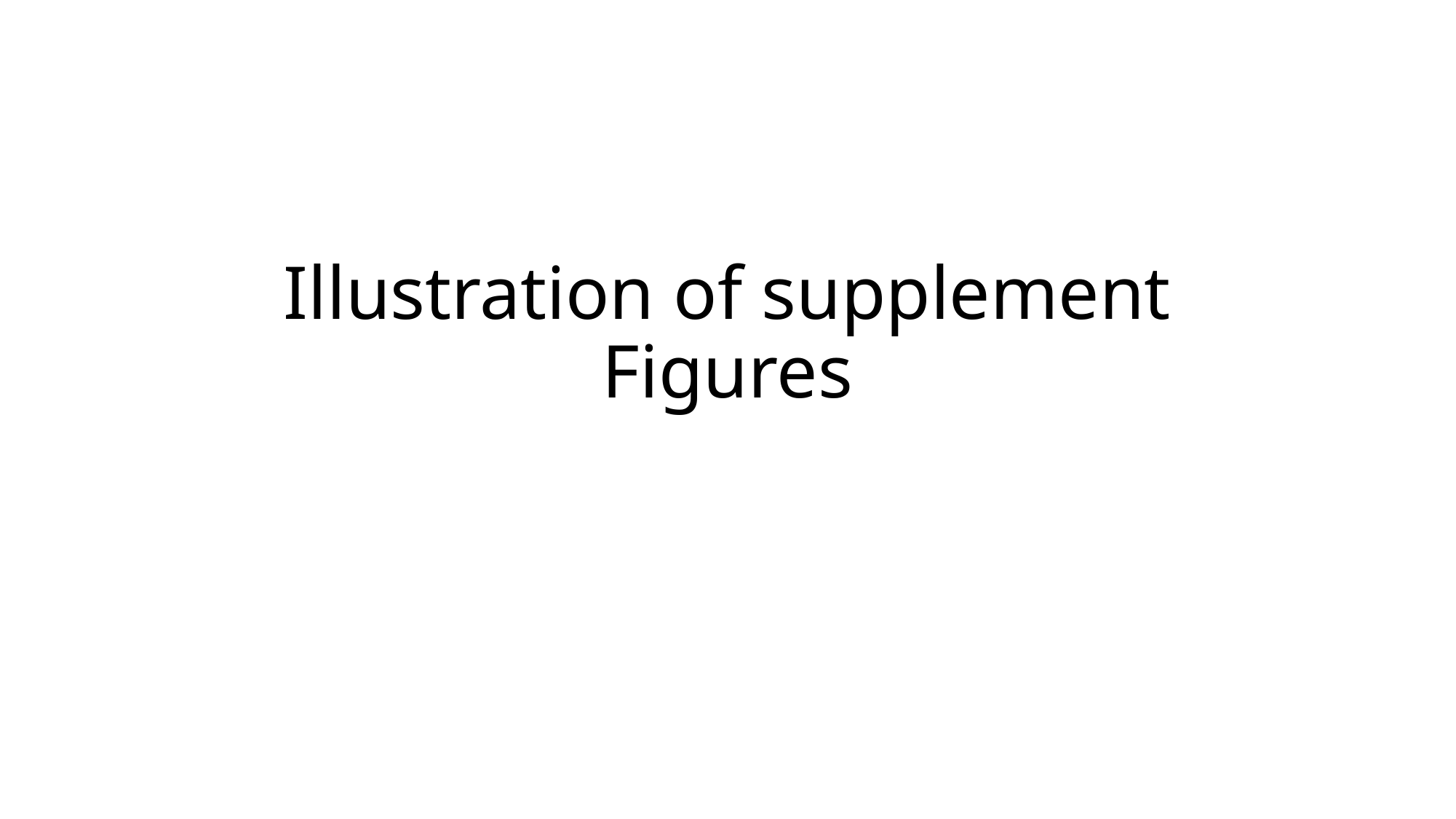

# Illustration of supplement Figures

## Slide 2
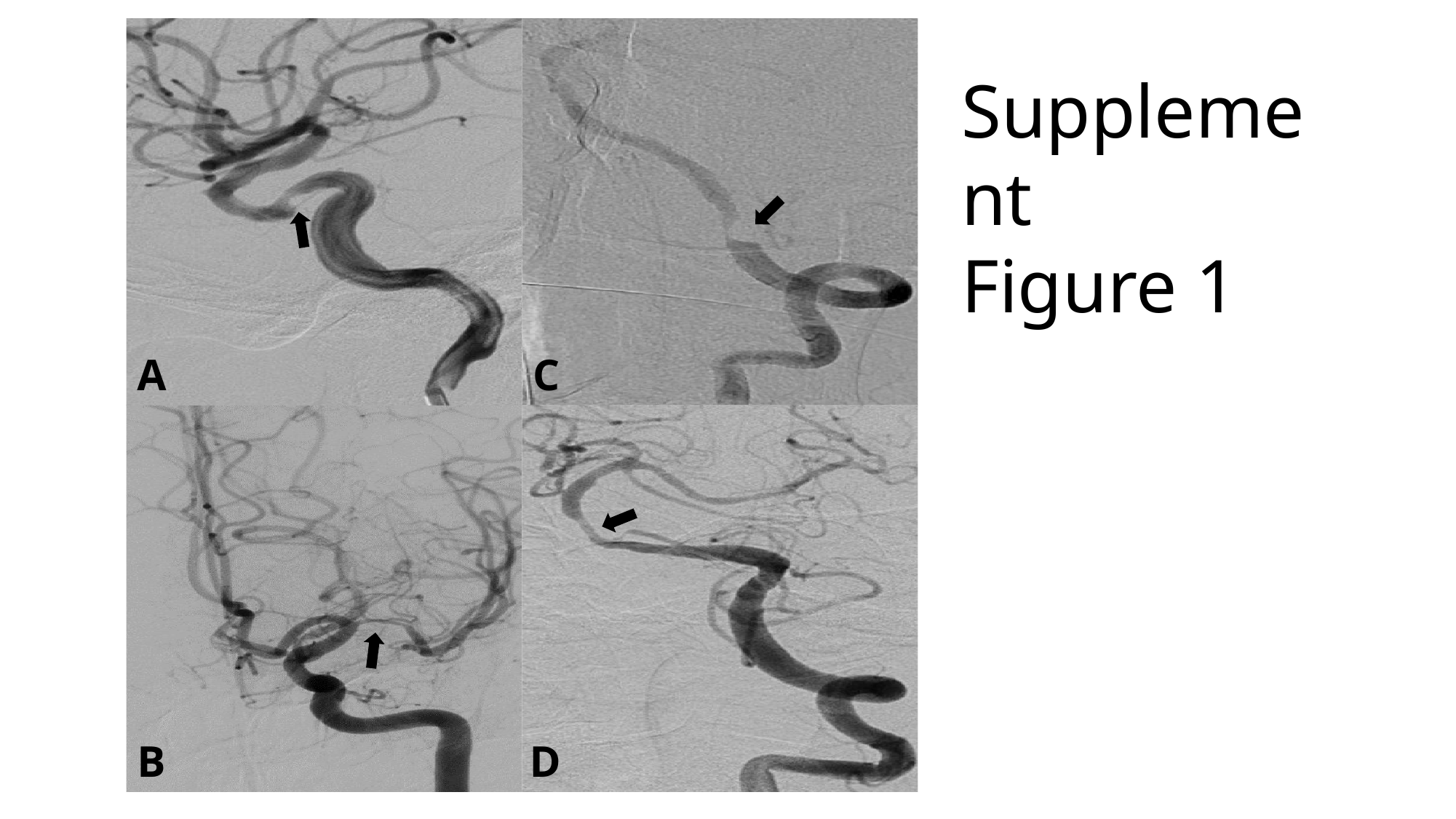

Supplement
Figure 1
A
C
B
D

## Slide 3
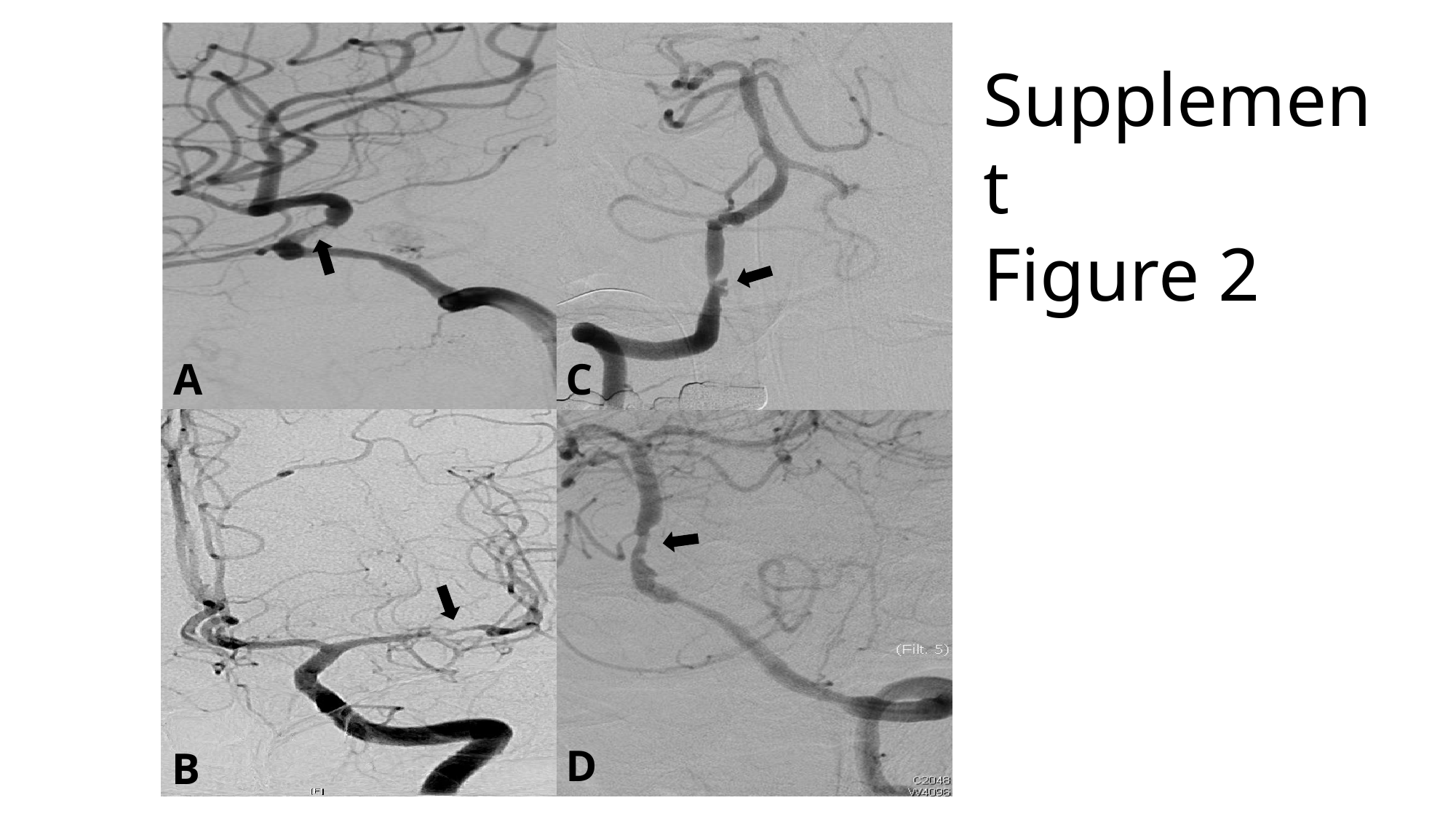

Supplement
Figure 2
A
C
D
B

## Slide 4
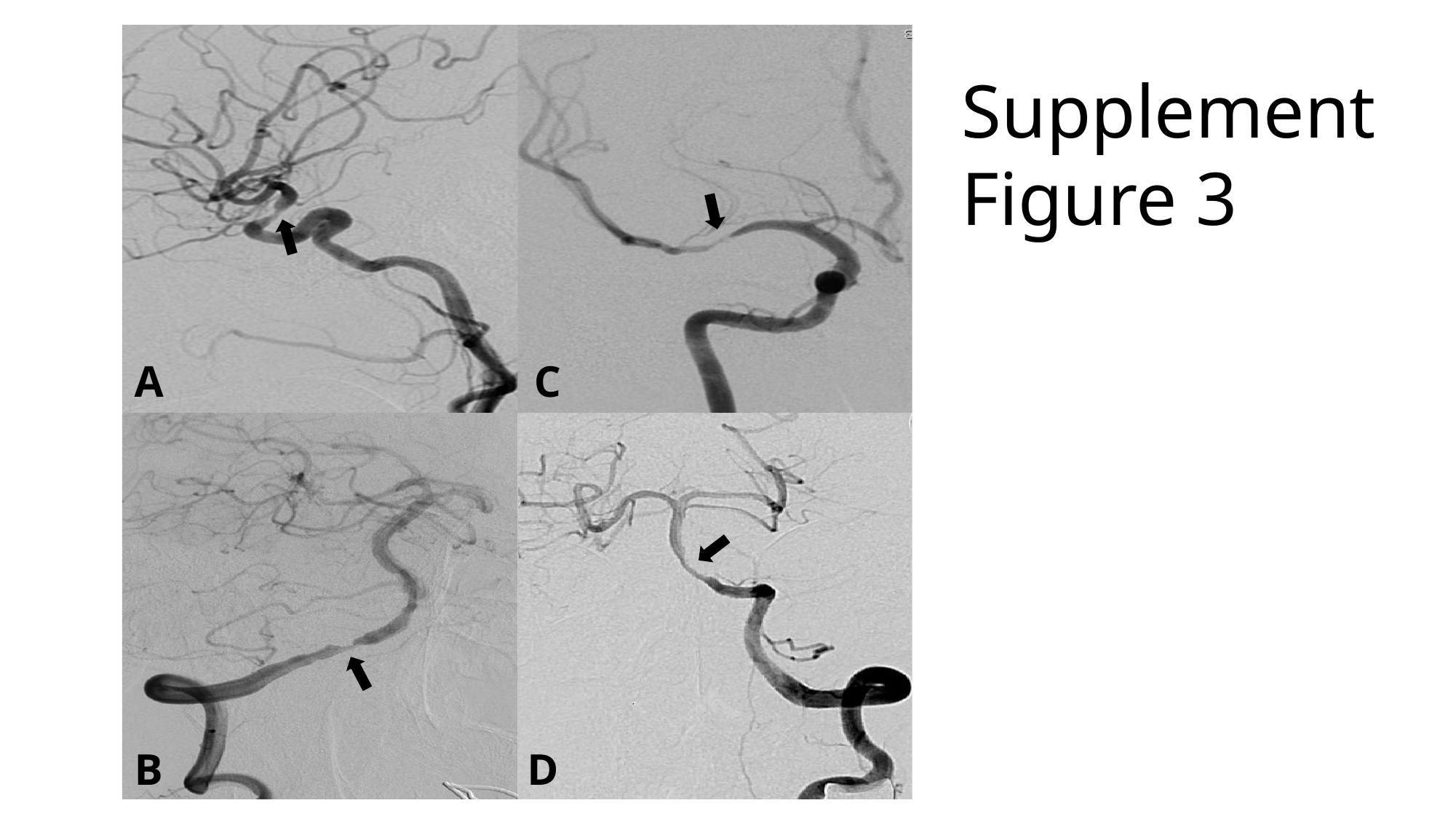

Supplement
Figure 3
A
C
D
B

## Slide 5
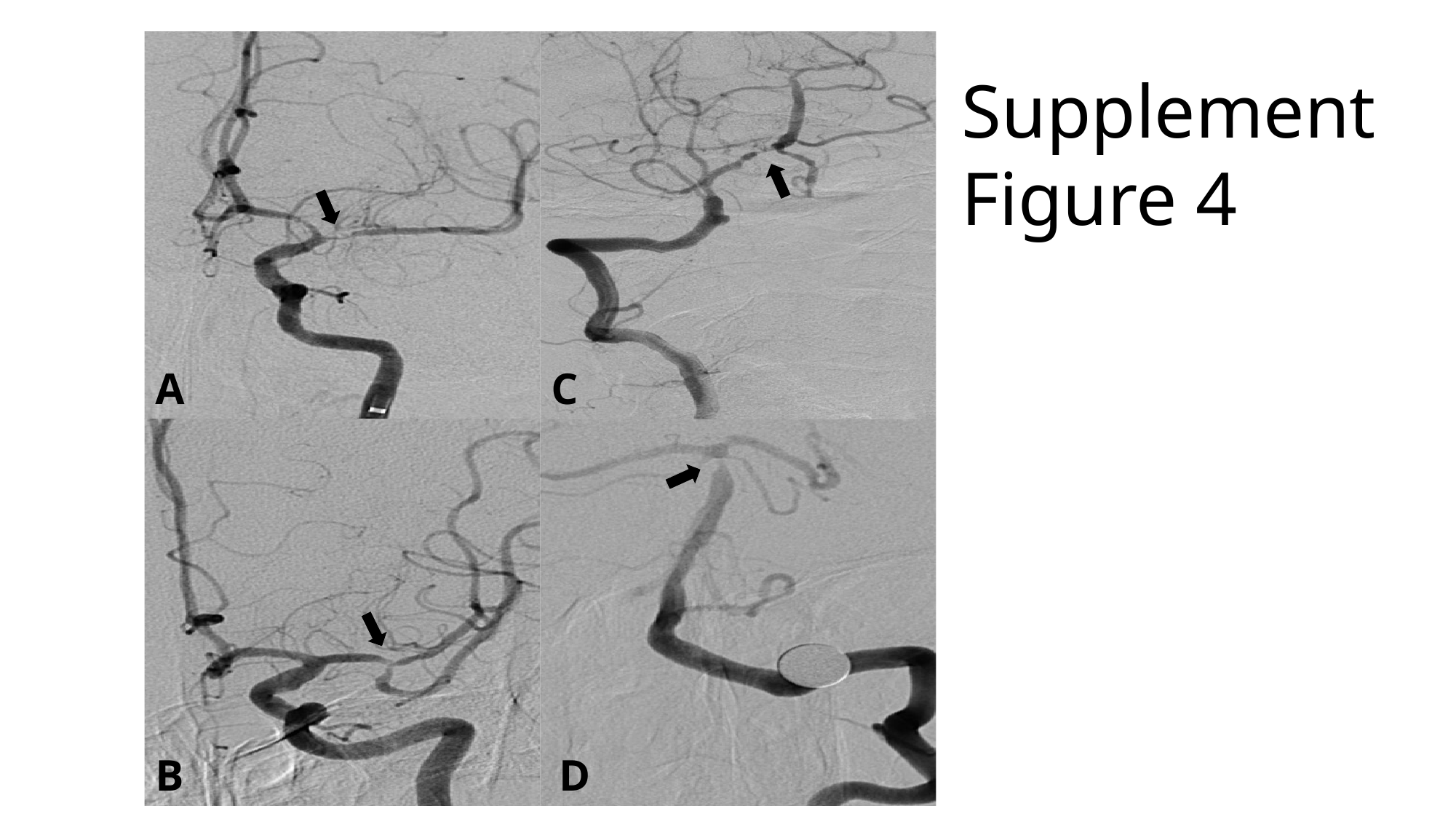

Supplement
Figure 4
A
C
B
D
